# Supplementary material for: Epigenome-wide meta-analysis of DNA methylation differences in prefrontal cortex implicates the immune processes in Alzheimer’s disease
Source: Nat Commun. 2020 Nov 30;11:6114. doi: 10.1038/s41467-020-19791-w (PMC7704686; doi:10.1038/s41467-020-19791-w)
Supplement: Supplementary file 1 — Supplementary Information [file 41467_2020_19791_MOESM1_ESM.pdf]

Supplementary Figure 1 Quantile-quantile (QQ) plots of observed and expected distributions of p-values in Gasparoni, London, Mount Sinai, and ROSMAP cohorts. λ is the genomic inflation factor, and λ_{bacon} is the genomic inflation factor estimated using the method of Iterson et al. (2017) (PMID: 28129774), as implemented in the *bacon* R package. Shading indicates 95% confidence intervals. Reference line in red indicates expected distribution of $-\log_{10}$(P-values) under the null hypothesis of no association.

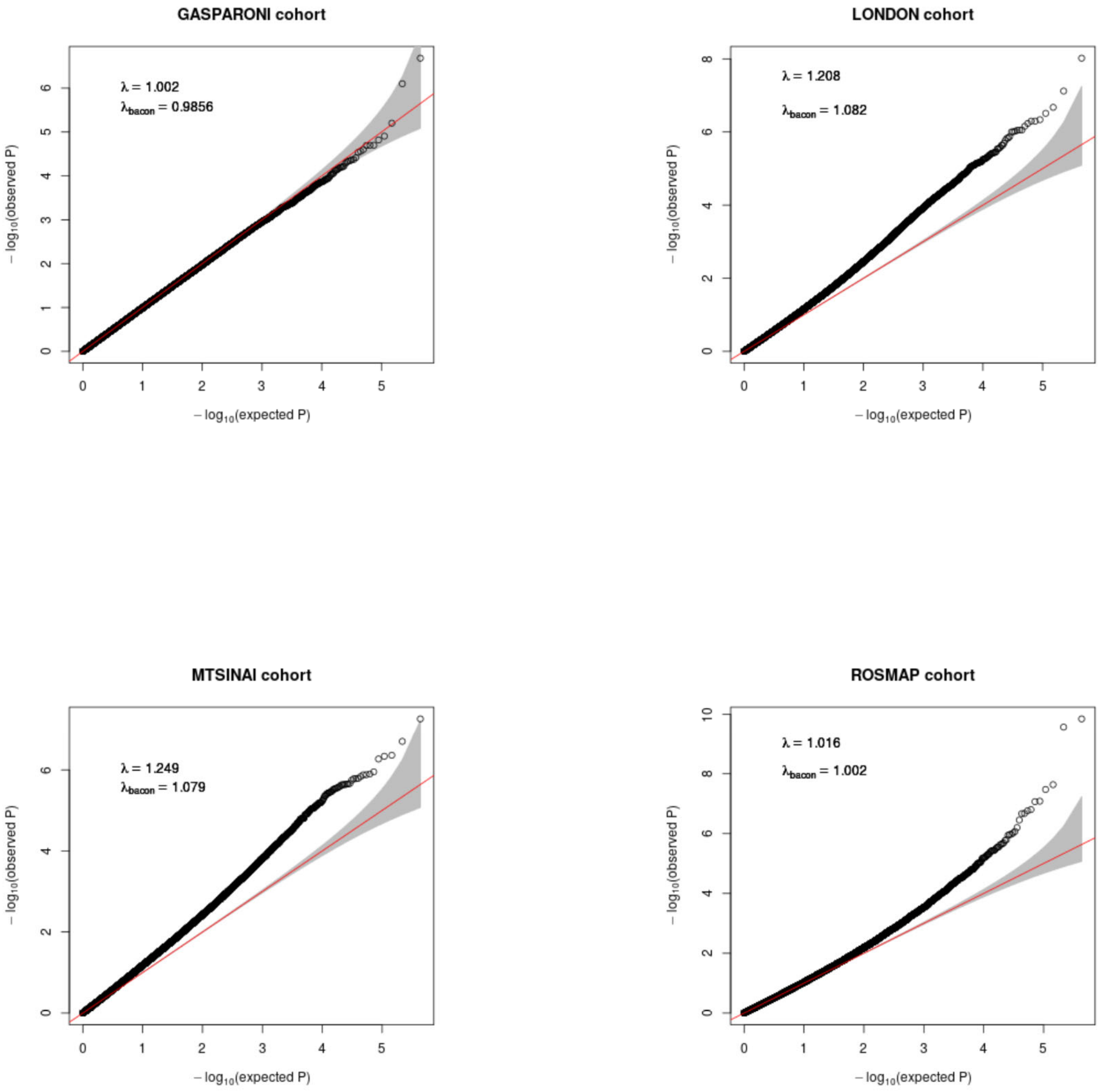

Supplementary Figure 2 Enrichment of CpGs significantly associated with the AD Braak stage in meta-analysis of individual CpGs at 5% FDR, after inflation correction by *bacon* method. A two-sided Fisher's test was used to determine over- or under-representation of the significant CpGs in various (A) genomic features and (B) chromatin states. *** indicates P-value < 0.001, ** indicates P-value < 0.01 and * indicates P-value < 0.05, uncorrected for multiple comparisons.

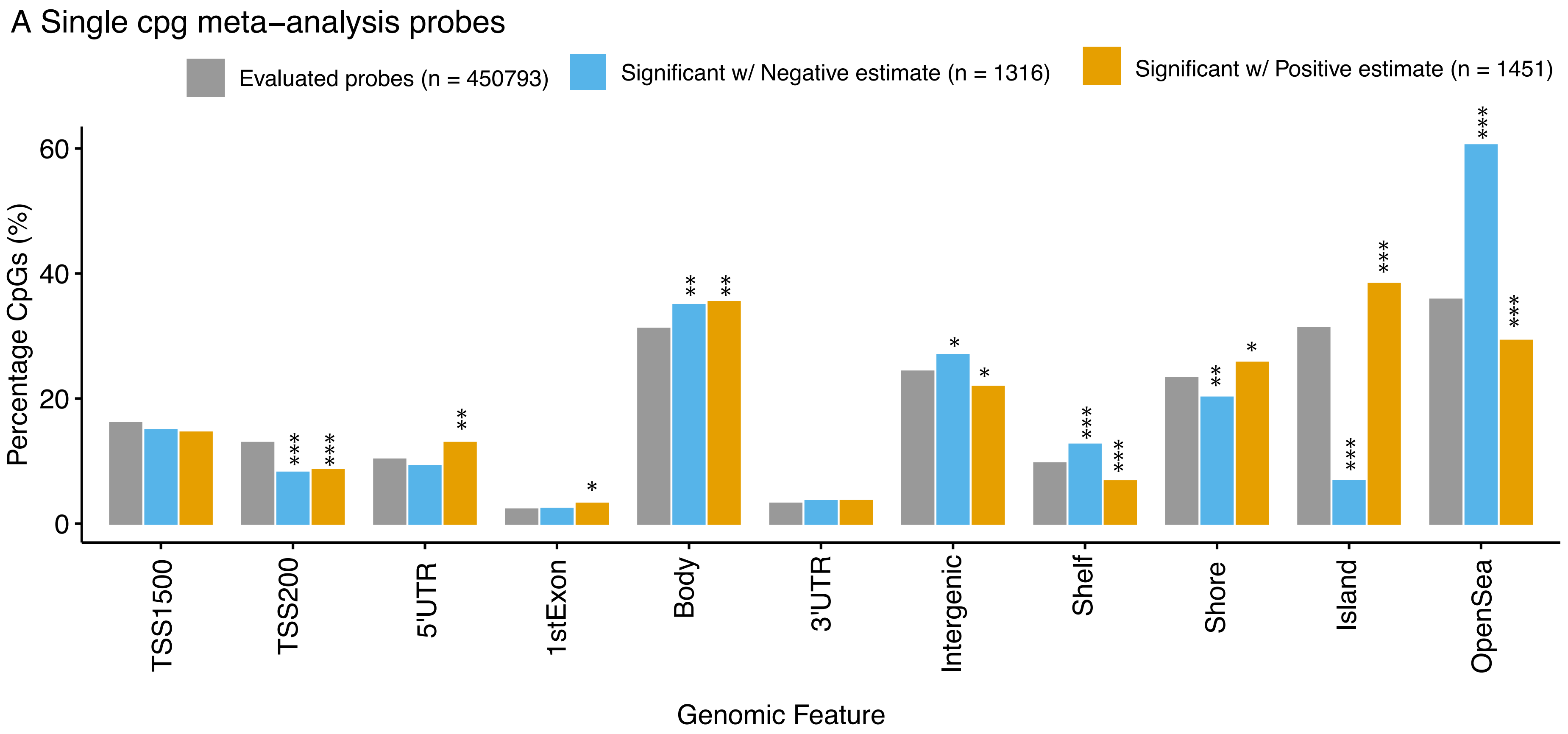

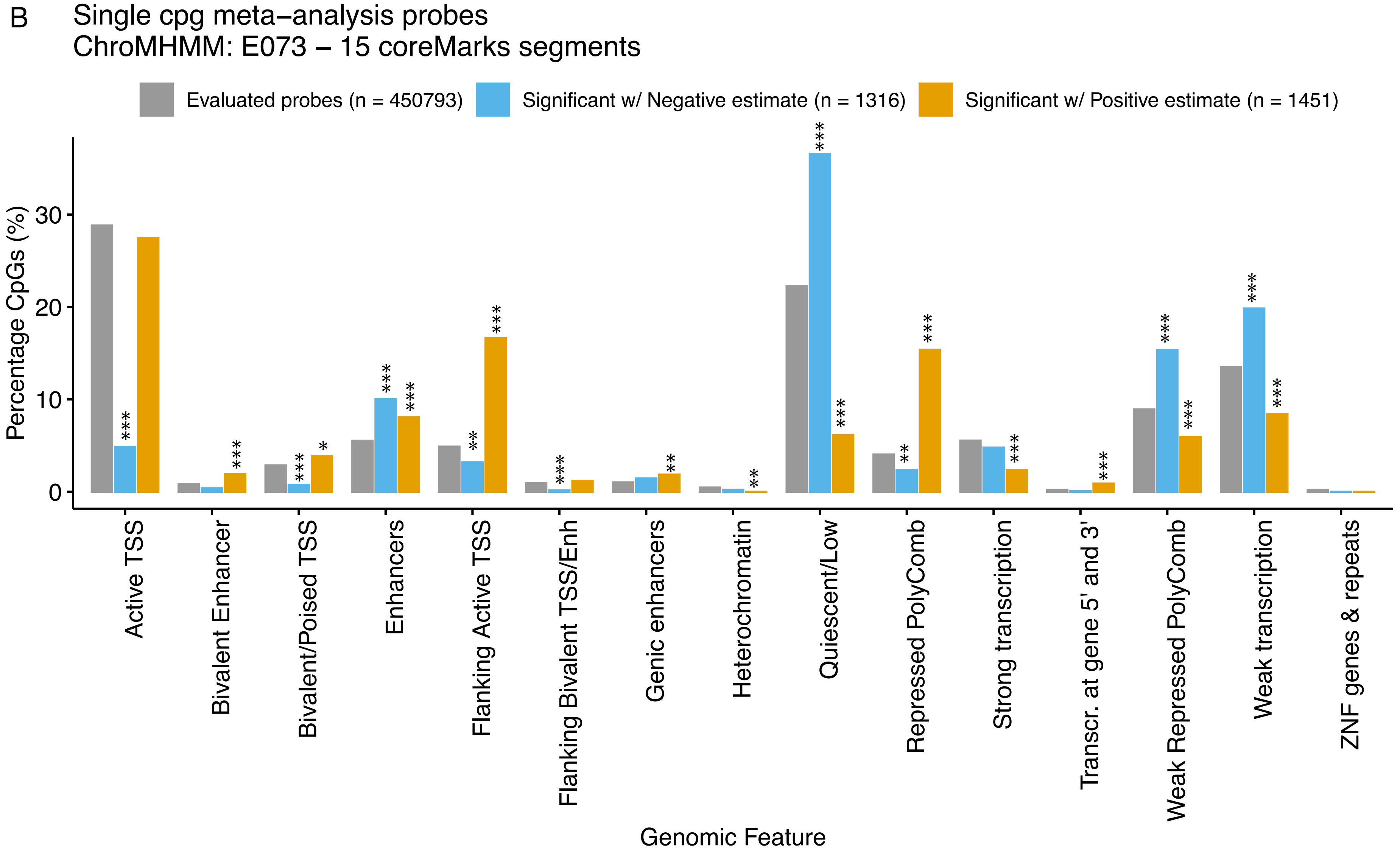

Supplementary Figure 3 Enrichment of CpGs significantly associated with AD Braak stage in meta-analysis of individual CpGs reaching genome-wide significance (2.4×10^{-7}), after inflation correction by *bacon* method. A two-sided Fisher's test was used to determine over- or under-representation of the significant CpGs in various (A) genomic features and (B) chromatin states. *** indicates P-value < 0.001, ** indicates P-value < 0.01 and * indicates P-value < 0.05, uncorrected for multiple comparisons.

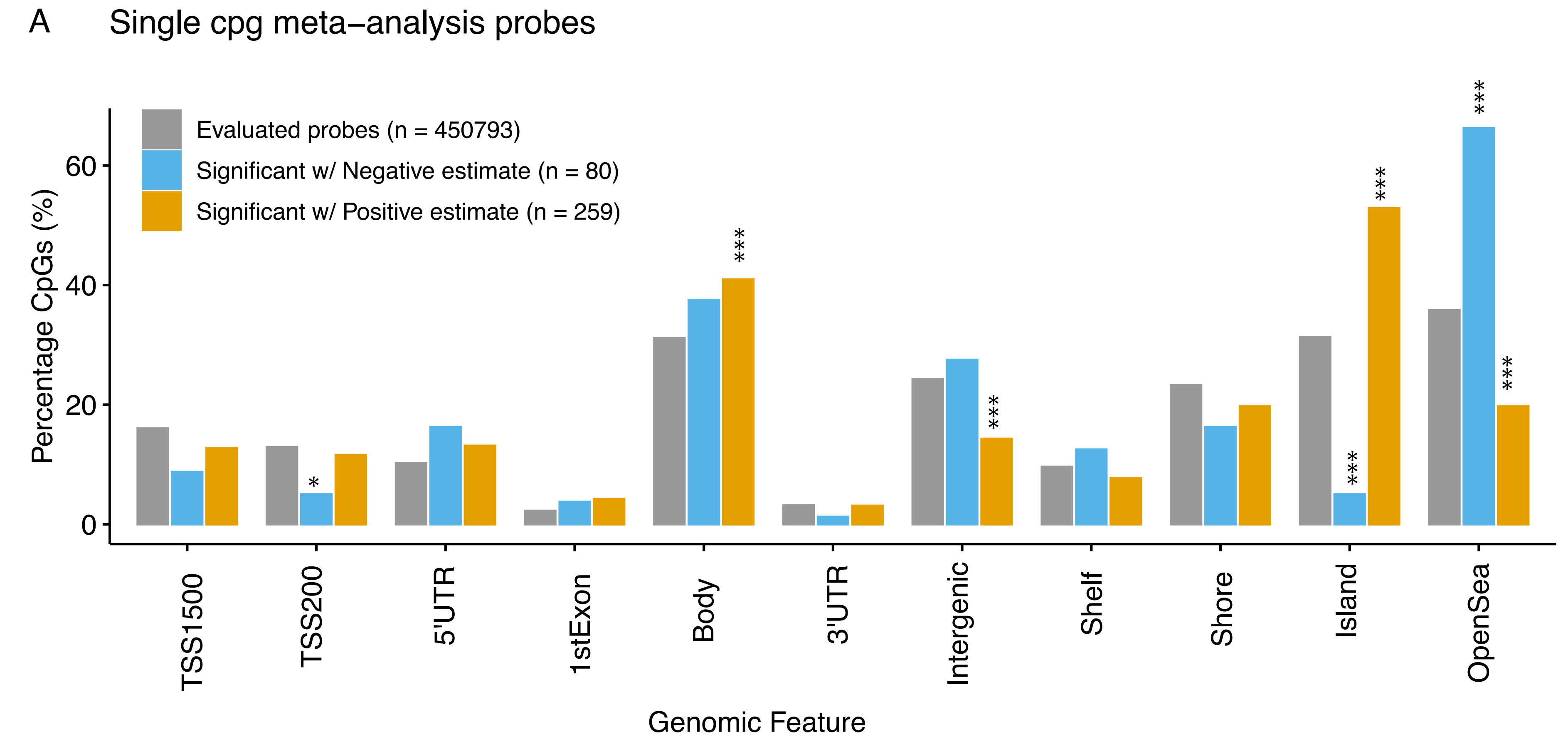

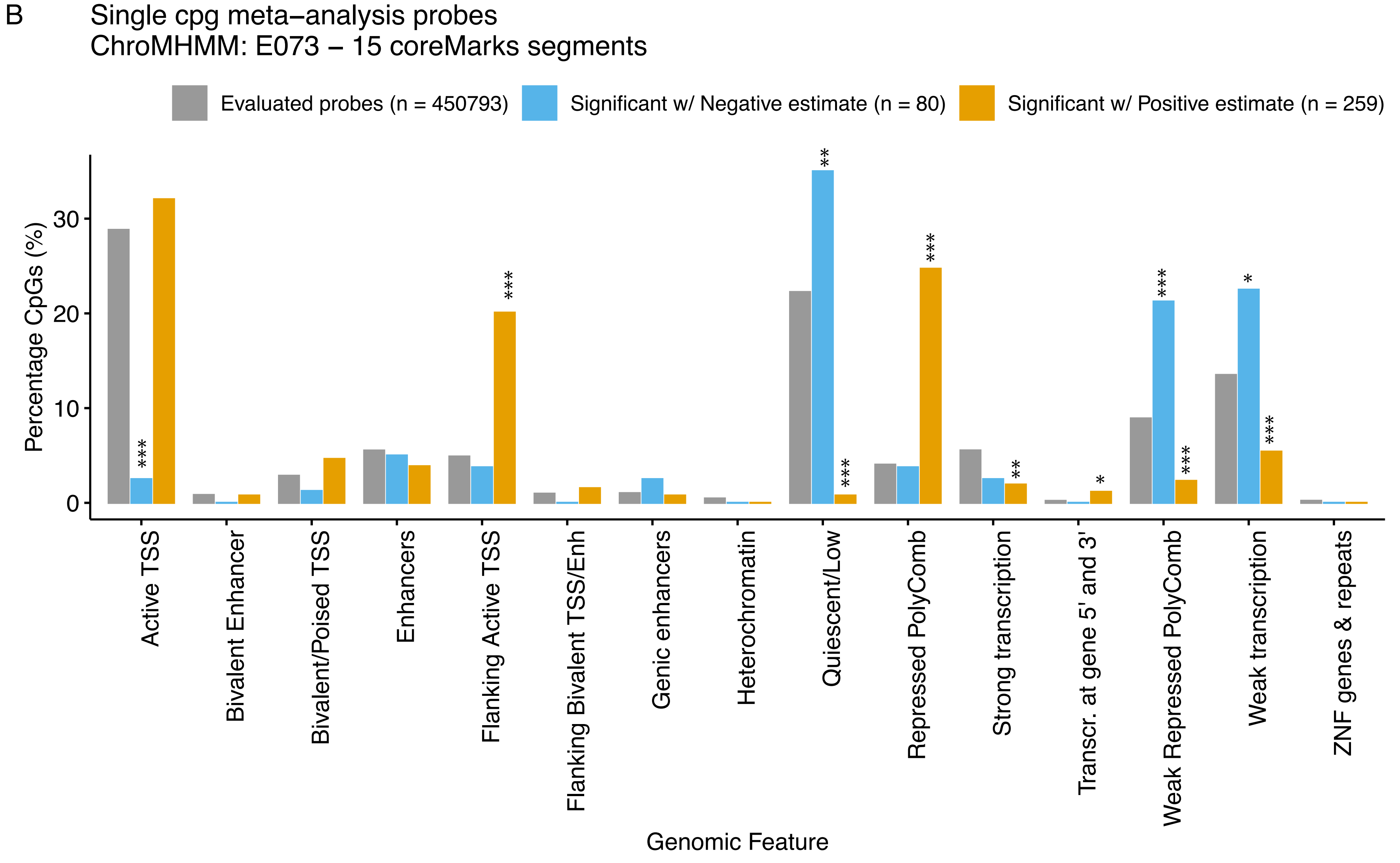

Supplementary Figure 4 Co-methylated regions (identified by coMethDMR) in the top 10 most significant DMRs in meta-analysis. Shown is the Spearman correlation of methylation M-values in pairs of probes within a co-methylated region identified by coMethDMR.

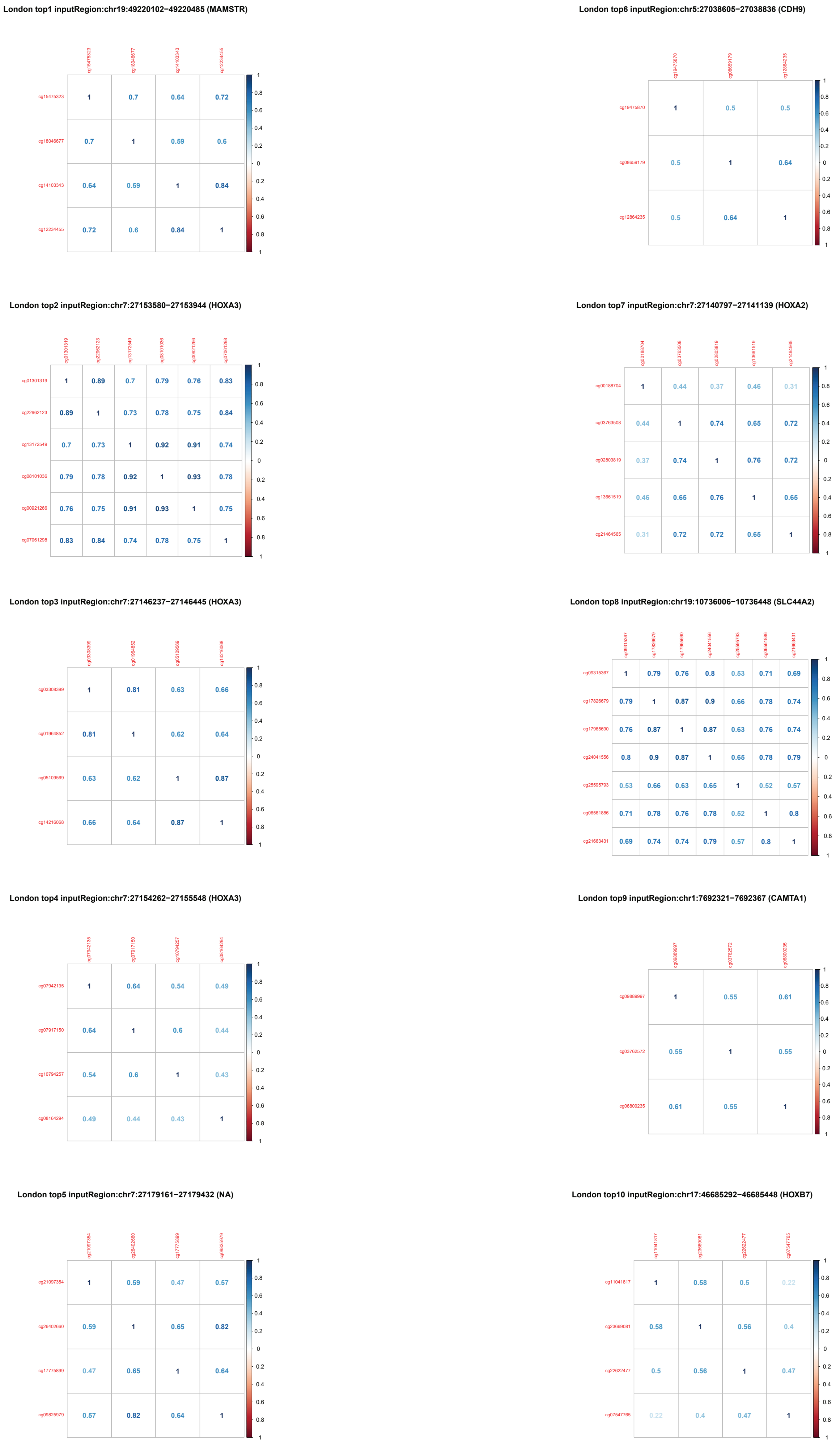

MtSinai top1 inputRegion:chr19:49220102−49220485 (MAMSTR)

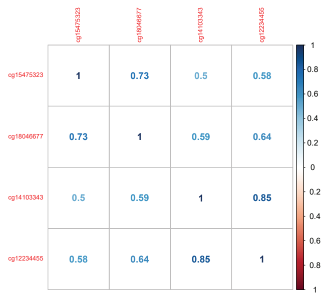

MtSinai top2 inputRegion:chr7:27153580−27153944 (HOXA3)

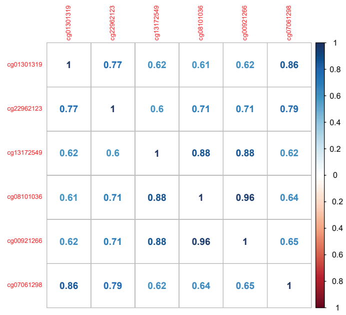

MtSinai top3 inputRegion:chr7:27146237−27146445 (HOXA3)

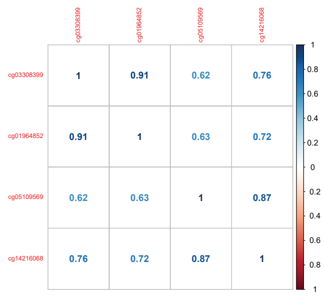

MtSinai top4 inputRegion:chr7:27154262−27155548 (HOXA3)

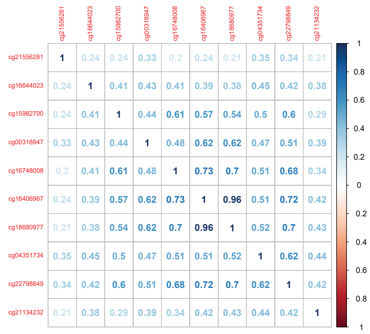

MtSinai top5 inputRegion:chr7:27179161−27179432 (NA)

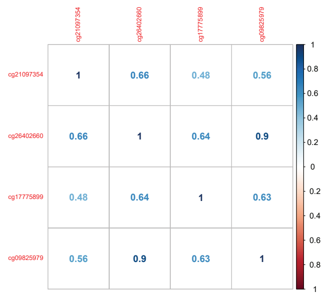

MtSinai top6 inputRegion:chr5:27038605−27038836 (CDH9)

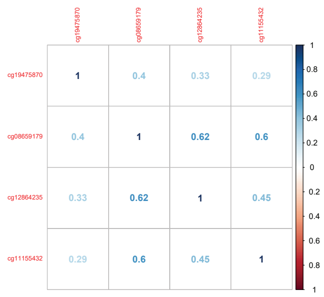

MtSinai top7 inputRegion:chr7:27140797−27141139 (HOXA2)

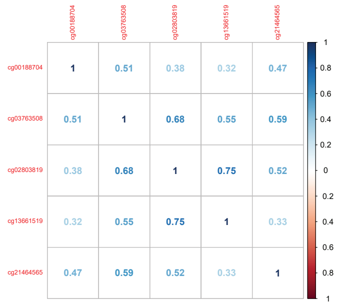

MtSinai top8 inputRegion:chr19:10736006−10736448 (SLC44A2)

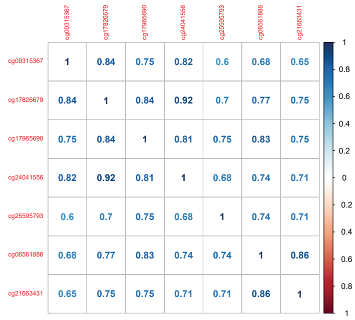

MtSinai top9 inputRegion:chr1:7692321−7692367 (CAMTA1)

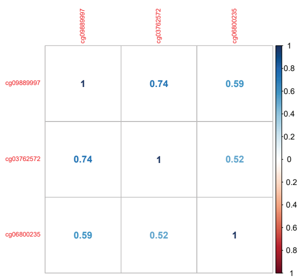

MtSinai top10 inputRegion:chr17:46685292−46685448 (HOXB7)

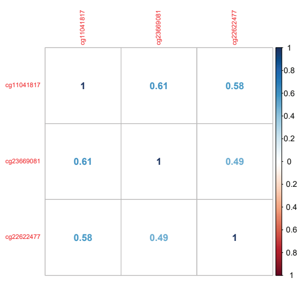

ROSMAP top1 inputRegion:chr19:49220102−49220485 (MAMSTR)

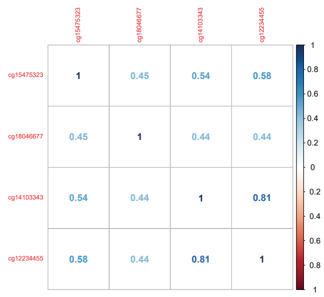

ROSMAP top6 inputRegion:chr5:27038605−27038836 (CDH9)

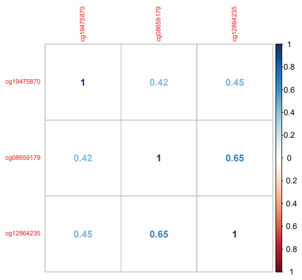

ROSMAP top2 inputRegion:chr7:27153580−27153944 (HOXA3)

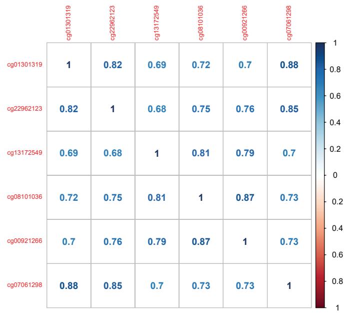

ROSMAP top7 inputRegion:chr7:27140797−27141139 (HOXA2)

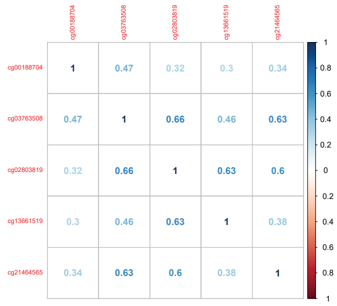

ROSMAP top3 inputRegion:chr7:27146237−27146445 (HOXA3)

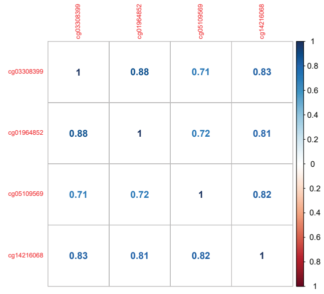

ROSMAP top8 inputRegion:chr19:10736006−10736448 (SLC44A2)

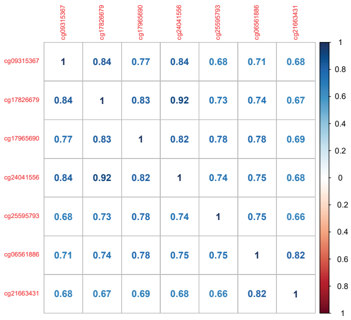

ROSMAP top4 inputRegion:chr7:27154262−27155548 (HOXA3)

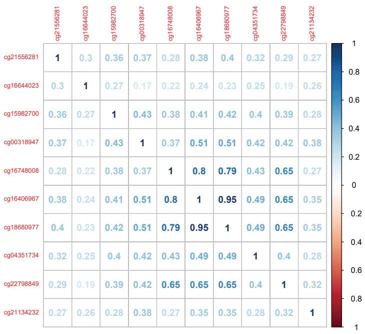

ROSMAP top9 inputRegion:chr1:7692321−7692367 (CAMTA1)

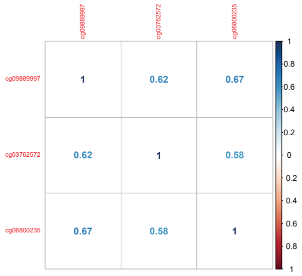

ROSMAP top5 inputRegion:chr7:27179161−27179432 (NA)

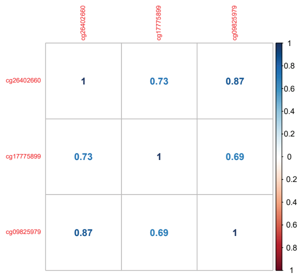

ROSMAP top10 inputRegion:chr17:46685292−46685448 (HOXB7)

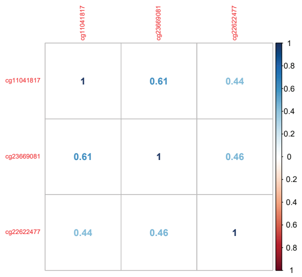

GASPARONI top1 inputRegion:chr19:49220102−49220485 (MAMSTR)

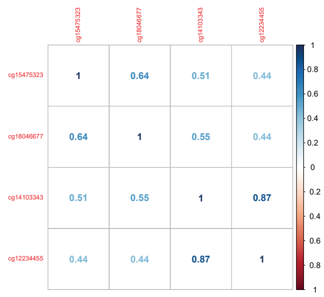

GASPARONI top2 inputRegion:chr7:27153580−27153944 (HOXA3)

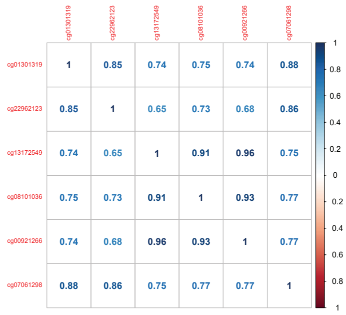

GASPARONI top3 inputRegion:chr7:27146237−27146445 (HOXA3)

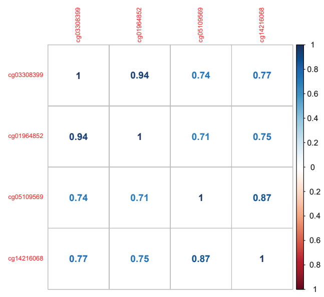

GASPARONI top4 inputRegion:chr7:27154262−27155548 (HOXA3)

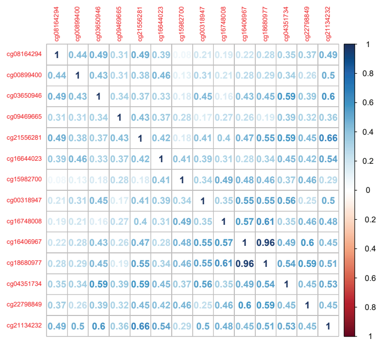

GASPARONI top5 inputRegion:chr7:27179161−27179432 (NA)

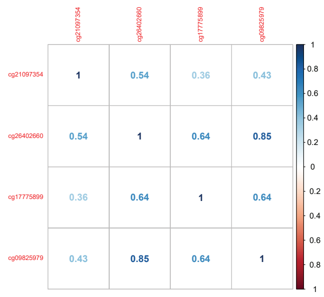

GASPARONI top6 inputRegion:chr5:27038605−27038836 (CDH9)

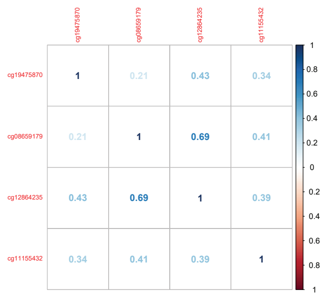

GASPARONI top7 inputRegion:chr7:27140797−27141139 (HOXA2)

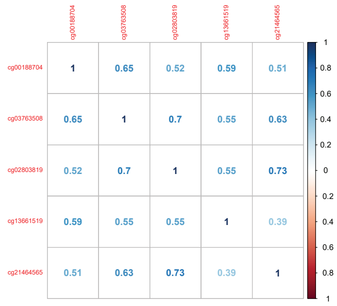

GASPARONI top8 inputRegion:chr19:10736006−10736448 (SLC44A2)

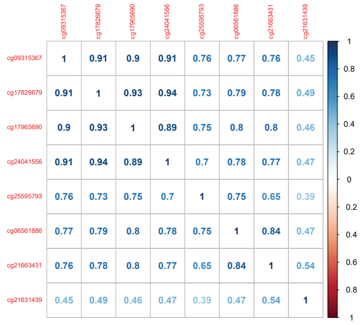

GASPARONI top9 inputRegion:chr1:7692321−7692367 (CAMTA1)

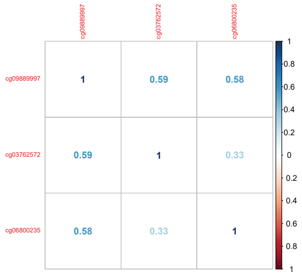

GASPARONI top10 inputRegion:chr17:46685292−46685448 (HOXB7)

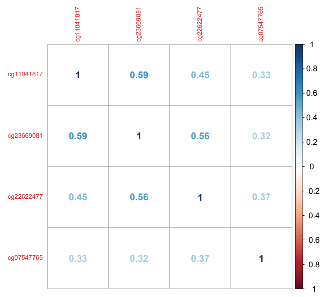

Supplementary Figure 5 Forest plots of meta-analysis effect sizes in the top 10 CpGs and top 10 DMRs across four brain samples cohorts (Gasparoni, London, Mt. Sinai, ROSMAP). Shown are effect sizes for the Braak stage in individual cohorts and meta-analysis.

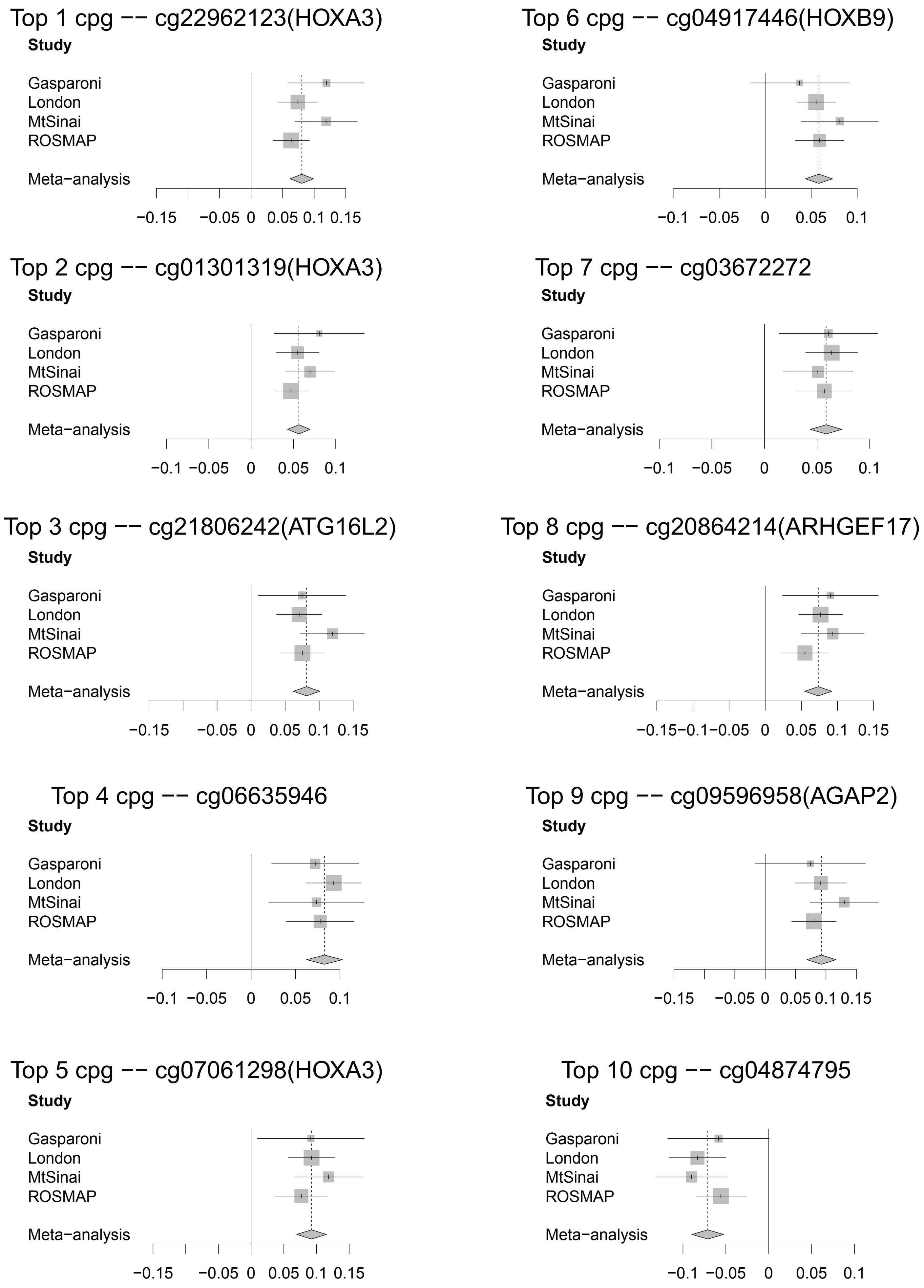

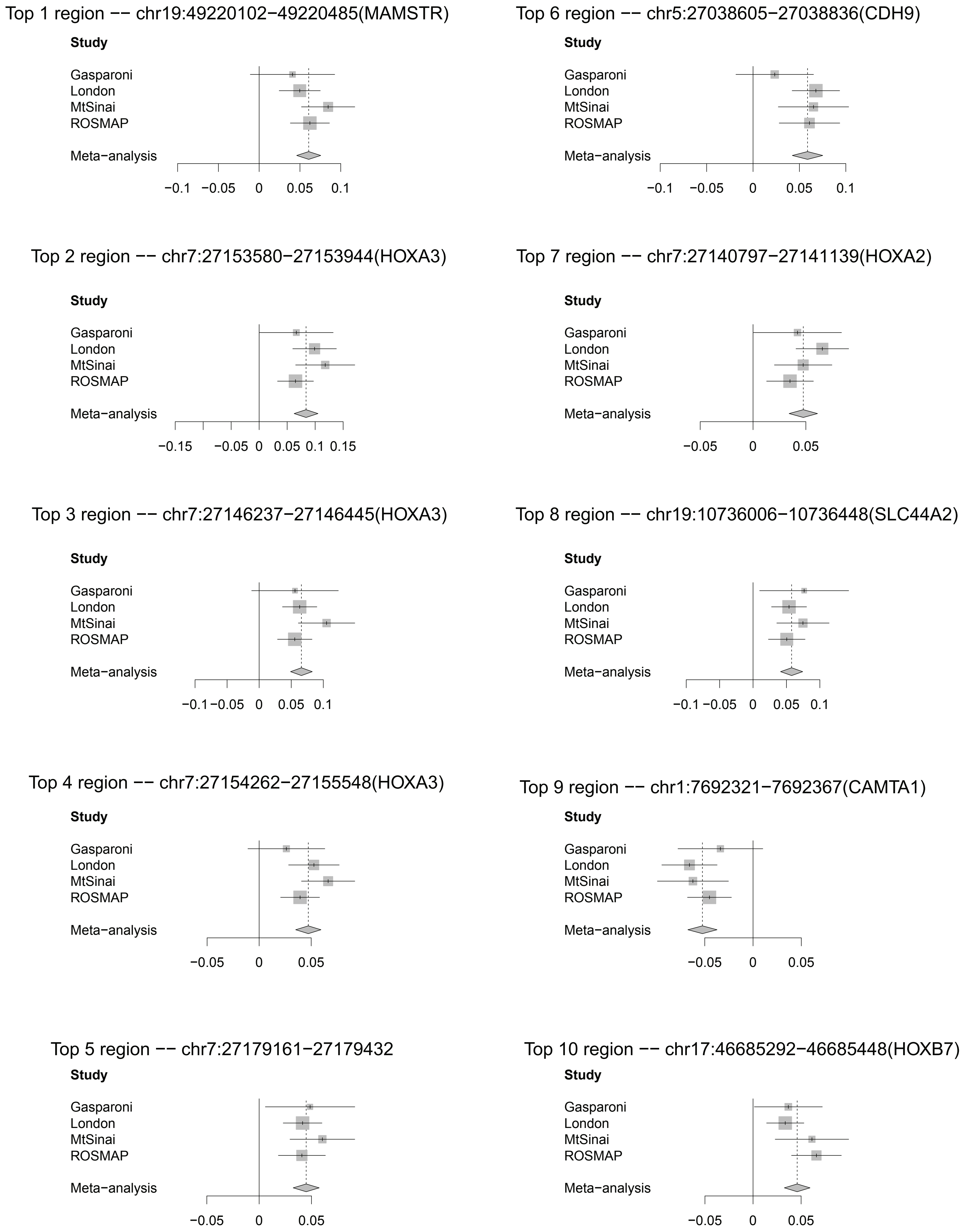
Top 1 region −− chr19:49220102−49220485(MAMSTR)
Study
Gasparoni
London
MtSinai
ROSMAP
Meta−analysis
−0.1 −0.05 0 0.05 0.1
Top 6 region −− chr5:27038605−27038836(CDH9)
Study
Gasparoni
London
MtSinai
ROSMAP
Meta−analysis
−0.1 −0.05 0 0.05 0.1
Top 2 region −− chr7:27153580−27153944(HOXA3)
Study
Gasparoni
London
MtSinai
ROSMAP
Meta−analysis
−0.15 −0.05 0 0.05 0.1 0.15
Top 7 region −− chr7:27140797−27141139(HOXA2)
Study
Gasparoni
London
MtSinai
ROSMAP
Meta−analysis
−0.05 0 0.05
Top 3 region −− chr7:27146237−27146445(HOXA3)
Study
Gasparoni
London
MtSinai
ROSMAP
Meta−analysis
−0.1 −0.05 0 0.05 0.1
Top 8 region −− chr19:10736006−10736448(SLC44A2)
Study
Gasparoni
London
MtSinai
ROSMAP
Meta−analysis
−0.1 −0.05 0 0.05 0.1
Top 4 region −− chr7:27154262−27155548(HOXA3)
Study
Gasparoni
London
MtSinai
ROSMAP
Meta−analysis
−0.05 0 0.05
Top 9 region −− chr1:7692321−7692367(CAMTA1)
Study
Gasparoni
London
MtSinai
ROSMAP
Meta−analysis
−0.05 0 0.05
Top 5 region −− chr7:27179161−27179432
Study
Gasparoni
London
MtSinai
ROSMAP
Meta−analysis
−0.05 0 0.05
Top 10 region −− chr17:46685292−46685448(HOXB7)
Study
Gasparoni
London
MtSinai
ROSMAP
Meta−analysis
−0.05 0 0.05
