# Supplementary material for: Epigenome-wide meta-analysis of DNA methylation differences in prefrontal cortex implicates the immune processes in Alzheimer’s disease
Source: Nat Commun. 2020 Nov 30;11:6114. doi: 10.1038/s41467-020-19791-w (PMC7704686; doi:10.1038/s41467-020-19791-w)
Supplement: Supplementary file 3 — Description of Additional Supplementary Files [file 41467_2020_19791_MOESM3_ESM.pdf]

Description of Additional Supplementary Files

Title: Supplementary Data 1
Description: A total of 3751 differentially methylated CpGs were significantly associated with the AD Braak stage at 5% FDR in the meta-analysis of four brain samples cohorts (Gasparoni, London, Mt. Sinai, ROSMAP).

Title: Supplementary Data 2
Description: A total of 119 co-methylated DMRs were significantly associated with AD Braak stage, which were identified by both coMethDMR (at 5% FDR) and comb-p (at 5% Sidak adjusted p-value) methods in meta-analysis of four brain samples cohorts (Gasparoni, London, Mount Sinai, ROSMAP).

Title: Supplementary Data 3
Description: Enrichment analysis of 119 FDR significant DMRs and 3751 FDR significant differentially methylated CpGs identified in meta-analysis in different types of genomic features. A two-sided Fisher's exact test was used to determine over- or under-representation of the significant CpGs or regions in each type of genomic feature.

Title: Supplementary Data 4
Description: Enrichment analysis of 119 FDR significant DMRs and 3751 FDR significant differentially methylated CpGs identified in meta-analysis in different types of chromatin states. A two-sided Fisher's exact test was used to determine over- or under-representation of the significant CpGs or regions in each type of chromatin state.

Title: Supplementary Data 5
Description: Enrichment of FDR significant differentially methylated CpGs in meta-analysis in binding sites of transcription factors and chromatin proteins assayed by ENCODE project. This analysis was performed using LOLA (Locus Overlap Analysis) software.

Title: Supplementary Data 6
Description: In samples matched by sex and age at death within the same cohort, a total of 151 differentially methylated CpGs were significantly associated with the AD Braak stage at 5% FDR in the meta-analysis of three brain samples cohorts (London, Mt. Sinai, ROSMAP).

Title: Supplementary Data 7
Description: In samples matched by sex and age at death within the same cohort, a total of 32 DMRs were significantly associated with AD Braak stage at 5% FDR in meta-analysis of three brain samples cohorts (London, Mt. Sinai, ROSMAP).

Title: Supplementary Data 8
Description: Comparison of the brain and blood DNA methylation levels using London cohort samples and BeCon software.

Title: Supplementary Data 9
Description: Association between methylation levels at Braak-associated DMRs with nearby genes. Each DMR is linked to genes located in the vicinity (± 250 kb). First, each DMR is summarized by the median of CpG methylation M-values over all CpGs mapped within the DMR. The median methylation M-values and normalized gene expression values are then adjusted for age at death, sex, cell type, and batch effects separately. Next, the residuals from these linear models are extracted. Finally, a separate linear model is used to test association between methylation residuals and gene expression residuals, adjusting for Braak stage.

Title: Supplementary Data 10
Description: Association between methylation levels at Braak-associated CpGs with nearby genes. Each CpG is linked to genes located in the vicinity (± 250 kb from the position of the CpG). The methylation M-values and normalized gene expression values are first adjusted for age at death, sex, cell type, and batch effects separately. Next, the residuals from these linear models are extracted. Finally, a separate linear model is used to test the association between methylation residuals and gene expression residuals, adjusting for Braak stage.

Title: Supplementary Data 11
Description: Among the 3751 FDR significant Braak-associated CpGs, 1010 CpGs had at least one corresponding mQTL in the prefrontal cortex brain samples. The mQTL analysis was performed using the ROSMAP cohort samples with matched genotype data and DNA methylation data for 688 samples. *cis* mQTLs located within 500kb from the CpGs were considered. The genotype data was imputed to HRC r1.1 reference panel and tested against AD status using logistic regression adjusting for age, sex, and first three PCs estimated from genotype data. AD status of the samples was determined using clinical consensus diagnosis of cognitive status at the time of death (cases: variable cogdx = 4, 5, controls: others).

Title: Supplementary Data 12
Description: Among the 119 FDR significant DMRs, 37 DMRs had at least one corresponding mQTL in the brain samples. The mQTL analysis was performed using the ROSMAP cohort samples with matched genotype data and DNA methylation data for 688 samples. *cis* mQTLs located within 500kb from the start or end of the DMRs were considered. The genotype data was imputed to HRC r1.1 reference panel and tested against AD status using logistic regression adjusting for age, sex, and first three PCs estimated from genotype data. AD status of the samples was determined using clinical consensus diagnosis of cognitive status at the time of death (cases: variable cogdx = 4, 5, controls: others).

Title: Supplementary Data 13
Description: Overlap of the 3751 FDR significant Braak-associated CpGs with AD GWAS loci (LDblockGRCh37) reported in Kunkle et al. (2019).

Title: Supplementary Data 14
Description: Bayesian co-localization of association signals from AD meta-analysis (Kunkle et al. (2019) PMID: 30820047) and ROSMAP mQTL study identified 2 GWAS nominated regions that included a single causal variant common to both traits (i.e. AD status and DNA methylation levels).

Title: Supplementary Data 15

Description: After *bacon*-correction, a total of 2767 differentially methylated CpGs were significantly associated with the AD Braak stage at 5% FDR, and a total of 339 CpGs reached genome-wide significance at 2.4×10^{-7}, in meta-analysis of four brain samples cohorts (Gasparoni, London, Mt. Sinai, ROSMAP).

Title: Supplementary Data 16

Description: Sensitivity analysis results for testing enrichment of *bacon*-corrected differentially methylated CpGs significantly associated with AD Braak stage in different genomic features. The p-values for single CpGs were subjected to *bacon* correction first, then the significant CpGs were identified as those that reached 5% FDR significance (*FDR significant CpGs;* n = 2767) or those that reached genome-wide significance at 2.4×10^{-7} (*genome-wide significant CpGs;* n = 339). A two-sided Fisher's exact test was used to determine over- or under-representation of the significant CpGs in each type of genomic feature.

Title: Supplementary Data 17

Description: Sensitivity analysis results for testing enrichment of *bacon*-corrected differentially methylated CpGs significantly associated with AD Braak stage in different chromatin states. The p-values for single CpGs were subjected to *bacon* correction first, then the significant CpGs were identified as those that reached 5% FDR significance (*FDR significant CpGs;* n = 2767) or those that reached genome-wide significance at 2.4×10^{-7}(*genome-wide significant CpGs;* n = 339). A two-sided Fisher's exact test was used to determine over- or under-representation of the significant CpGs in each type of chromatin state.

Title: Supplementary Data 18

Description: Sensitivity analysis results for testing enrichment of *bacon*-corrected differentially methylated CpGs significantly associated with AD Braak stage in binding sites of ENCODE transcription factors and chromatin proteins. The p-values for single CpGs were subjected to *bacon* correction first, then the significant CpGs were identified as those that reached 5% FDR significance (*fdr significant CpGs;* n = 2767). This analysis was performed using LOLA (Locus Overlap Analysis) software.

Title: Supplementary Data 19

Description: Sensitivity analysis results for gene set enrichment analysis of significant methylation differences associated with the AD Braak stage identified in meta-analysis. The p-values for single CpGs were subjected to *bacon* correction first, then the significant CpGs were identified as those that reached 5% FDR significance after *bacon* correction.

Title: Supplementary Data 20

Description: Sensitivity analysis results for gene set enrichment analysis of significant methylation differences associated with the AD Braak stage identified in meta-analysis. The p-values for single CpGs were subjected to *bacon* correction first, then the significant CpGs were identified as those that reached genome-wide significance after *bacon* correction.

Title: Supplementary Data 21

Description: Single CpG analysis results using QN.BMIQ pre-processing pipeline. Shown are p-values for testing association between methylation levels and Braak stage, adjusting for age, sex, and neuron proportions for London cohort samples (London_pval) and Mount Sinai cohort samples (MtSinai_pval).

These results are similar to those in Table 1 of Smith et al. (2018) (PMID: 29550519), analyzed using *dasen* analysis pipeline (PMID: 23631413).

Title: Supplementary Data 22
Description: Average gene expression levels for PRC2 target genes by AD stages in ROSMAP samples. The PRC2 target genes were identified in Title: Supplementary Datas 3 and 6 of Schimmelmann et al. (2016) (PMID: 27526204).

Title: Supplementary Data 23
Description: Quality control (QC) information on DNA methylation samples and probes for each cohort contributing to this meta-analysis.

Title: Supplementary Data 24
Description: Enrichment analysis results using mixed effects model (with random chromosomes effects to model correlations between CpGs) are similar to those from Fisher's exact test. The significant CpGs were identified as those reaching 5% FDR significance. A logistic mixed effects regression model was used to test the association between the type of genomic region (e.g. isCpGisland = “yes” or “no”) and the significance of the CpG (e.g. isSignificant = “yes” or “no”). Random effects for each chromosome were also included in this model to account for correlations between CpGs within the same chromosome. For comparison, enrichment analysis results using Fisher’s exact test from Title: Supplementary Datas 3 and 4 are also included.
