# Supplementary material for: Epigenome-wide meta-analysis of DNA methylation differences in prefrontal cortex implicates the immune processes in Alzheimer’s disease
Source: Nat Commun. 2020 Nov 30;11:6114. doi: 10.1038/s41467-020-19791-w (PMC7704686; doi:10.1038/s41467-020-19791-w)
Supplement: Supplementary file 5 — Reporting Summary [file 41467_2020_19791_MOESM5_ESM.pdf]

nature research

Corresponding author(s): Lily Wang

Last updated by author(s): 9/23/2020

Reporting Summary

Nature Research wishes to improve the reproducibility of the work that we publish. This form provides structure for consistency and transparency in reporting. For further information on Nature Research policies, see our Editorial Policies and the Editorial Policy Checklist.

Statistics

For all statistical analyses, confirm that the following items are present in the figure legend, table legend, main text, or Methods section.

n/a	Confirmed	
☐	☒	The exact sample size (n) for each experimental group/condition, given as a discrete number and unit of measurement
☐	☒	A statement on whether measurements were taken from distinct samples or whether the same sample was measured repeatedly
☐	☒	The statistical test(s) used AND whether they are one- or two-sided<br>*Only common tests should be described solely by name; describe more complex techniques in the Methods section.*
☐	☒	A description of all covariates tested
☐	☒	A description of any assumptions or corrections, such as tests of normality and adjustment for multiple comparisons
☐	☒	A full description of the statistical parameters including central tendency (e.g. means) or other basic estimates (e.g. regression coefficient) AND variation (e.g. standard deviation) or associated estimates of uncertainty (e.g. confidence intervals)
☐	☒	For null hypothesis testing, the test statistic (e.g. F, t, r) with confidence intervals, effect sizes, degrees of freedom and P value noted<br>*Give P values as exact values whenever suitable.*
☐	☒	For Bayesian analysis, information on the choice of priors and Markov chain Monte Carlo settings
☐	☒	For hierarchical and complex designs, identification of the appropriate level for tests and full reporting of outcomes
☐	☒	Estimates of effect sizes (e.g. Cohen's d, Pearson's r), indicating how they were calculated

Our web collection on statistics for biologists contains articles on many of the points above.

Software and code

Policy information about availability of computer code

Data collection	All analyses were performed using the R software (https://www.r-project.org/; version 3.6), Python software (version 2.7.12), SAS (version 9.4) and PLINK (version 2). The scripts used for all analyses in this study can be accessed at https://github.com/TransBioInfoLab/ad-meta-analysis. The version number of the software used can be accessed at end of each individual script under "Session information".
Data analysis	All analyses were performed using the R software (https://www.r-project.org/; version 3.6), Python software (version 2.7.12), SAS (version 9.4) and PLINK (version 2). The scripts used for all analyses in this study can be accessed at https://github.com/TransBioInfoLab/ad-meta-analysis. The version number of the software used can be accessed at end of each individual script under "Session information".

For manuscripts utilizing custom algorithms or software that are central to the research but not yet described in published literature, software must be made available to editors and reviewers. We strongly encourage code deposition in a community repository (e.g. GitHub). See the Nature Research guidelines for submitting code & software for further information.

Data

Policy information about availability of data

All manuscripts must include a data availability statement. This statement should provide the following information, where applicable:

- Accession codes, unique identifiers, or web links for publicly available datasets
- A list of figures that have associated raw data
- A description of any restrictions on data availability

Information on source data are provided with this paper, all datasets used in this study are publicly available as described in Table 1 and Methods. The ROSMAP, Mt. Sinai, London and Gasparoni datasets can be accessed from GEO (accessions GSE80970, GSE59685, GSE66351) and Synapse (accession syn3157275).

Field-specific reporting

Please select the one below that is the best fit for your research. If you are not sure, read the appropriate sections before making your selection.

☒ Life sciences ☐ Behavioural & social sciences ☐ Ecological, evolutionary & environmental sciences

For a reference copy of the document with all sections, see nature.com/documents/nr-reporting-summary-flat.pdf

Life sciences study design

All studies must disclose on these points even when the disclosure is negative.

Sample size	In this study, we meta-analyzed publicly available datasets, therefore, sample size were determined by the previous studies (PMIDs: 25129077, 25129075, 30045751, 29550519).
Data exclusions	We excluded samples that did not pass our quality control pre-processing pipeline. More specifically, quality control for samples included restricting our analysis to samples with good bisulfite conversion efficiency (i.e., ≥ 88%) and principal component analysis (PCA). More specifically, PCA was performed using the 50,000 most variable CpGs for each cohort. Samples that were within ± 3 standard deviations from the mean of PC1 and PC2 were selected to be included in the final sample set.
Replication	We did not have replication cohort for this study.
Randomization	In all our analyses, including integrative analysis of DNA methylation with gene expression or genetic variants, we adjusted for potential confounding effects including age at death, sex, cell types and batch effects.
Blinding	Since we used data generated by previous studies and we did not collect any new data, blinding is not relevant to this study.

Reporting for specific materials, systems and methods

We require information from authors about some types of materials, experimental systems and methods used in many studies. Here, indicate whether each material, system or method listed is relevant to your study. If you are not sure if a list item applies to your research, read the appropriate section before selecting a response.

Materials & experimental systems

n/a	Involved in the study	
☒	☐	Antibodies
☒	☐	Eukaryotic cell lines
☒	☐	Palaeontology and archaeology
☒	☐	Animals and other organisms
☒	☐	Human research participants
☒	☐	Clinical data
☒	☐	Dual use research of concern

Methods

n/a	Involved in the study	
☒	☐	ChIP-seq
☒	☐	Flow cytometry
☒	☐	MRI-based neuroimaging
